# Predictive value of MGMT promoter methylation on the survival of TMZ treated *IDH*-mutant glioblastoma

**DOI:** 10.20892/j.issn.2095-3941.2020.0179

**Published:** 2021-02-15

**Authors:** Ruichao Chai, Guanzhang Li, Yuqing Liu, Kenan Zhang, Zheng Zhao, Fan Wu, Yuzhou Chang, Bo Pang, Jingjun Li, Yangfang Li, Tao Jiang, Yongzhi Wang

**Affiliations:** 1 Department of Molecular Neuropathology, Beijing Neurosurgical Institute; Chinese Glioma Genome Atlas Network (CGGA), Capital Medical University, Beijing 100070, China; 2 Department of Neurosurgery, Beijing Tiantan Hospital, Capital Medical University, Beijing 100070, China

**Keywords:** Glioblastoma, O6methylguanine-DNA methyltransferase, isocitrate dehydrogenase, temozolomide, pyrosequencing

## Abstract

**Objective:**

O6methylguanine-DNA methyltransferase (*MGMT*) promoter methylation is a biomarker widely used to predict the sensitivity of *IDH*-wildtype glioblastoma to temozolomide therapy. Given that the *IDH* status has critical effects on the survival and epigenetic features of glioblastoma, we aimed to assess the role of *MGMT* promoter methylation in *IDH*-mutant glioblastoma.

**Methods:**

This study included 187* IDH*-mutant glioblastomas and used 173 *IDH*-wildtype glioblastomas for comparison. Kaplan-Meier curves and multivariate Cox regression were used to study the predictive effects.

**Results:**

Compared with *IDH*-wildtype glioblastomas, *IDH*-mutant glioblastomas showed significantly higher (*P* < 0.0001) *MGMT* promoter methylation. We demonstrated that *MGMT* promoter methylation status, as determined by a high cutoff value (≥30%) in pyrosequencing, could be used to significantly stratify the survival of 50 *IDH*-mutant glioblastomas receiving temozolomide therapy (cohort A); this result was validated in another cohort of 25 *IDH*-mutant glioblastomas (cohort B). The median progression-free survival and median overall survival in cohort A were 9.33 and 13.76 months for unmethylated cases, and 18.37 and 41.61 months for methylated cases, and in cohort B were 6.97 and 9.10 months for unmethylated cases, and 23.40 and 26.40 months for methylated cases. In addition, we confirmed that the *MGMT* promoter methylation was significantly (*P* = 0.0001) correlated with longer OS in *IDH*-mutant patients with GBM, independently of age, gender distribution, tumor type (primary or recurrent/secondary), and the extent of resection.

**Conclusions:**

*MGMT* promoter methylation has predictive value in *IDH*-mutant glioblastoma, but its cutoff value should be higher than that for *IDH*-wildtype glioblastoma.

## Introduction

The alkylating agent temozolomide (TMZ) is the first-line chemotherapy drug for glioma, the most common malignant primary brain tumor in adults^1–5^. Glioblastoma (GBM, WHO grade IV), the most aggressive glioma, has a median survival rate of 14–16 months despite intensive treatment including neurosurgical resection, concurrent radiotherapy and TMZ therapy, and adjuvant TMZ treatment for several cycles^1,2,6–8^. GBM are classified according to whether they express the wildtype or mutant isocitrate dehydrogenase (*IDH*) gene; more than 85% of all GBM have wildtype *IDH*^9,10^. The prognosis of *IDH*-wildtype cases is poorer than that of IDH-mutant cases, and the genetic, epigenetic, and clinical features differ between *IDH*-wildtype and *IDH*-mutant GBM^10–17^. On the basis of these findings, the Consortium to Inform Molecular and Practical Approaches to CNS Tumor Taxonomy (cIMPACT-NOW) update 5 suggests that *IDH*-mutant GBM should be denoted astrocytoma, *IDH*-mutant, grade 4, but notes that this change in this terminology may be viewed as controversial and will require further discussion in context of the next WHO classification^11^. Therefore, we have used the terminology of *IDH*-mutant GBM in this study.

O6-methylguanine-DNA methyltransferase (*MGMT*) is a DNA repair enzyme that can rapidly reverse alkylation at the O6 position with its own irreversible consumption^4^. The expression level of MGMT strongly depends on the methylation level of its promoter region^4,18^. In a series of clinical trials, promoter methylation of *MGMT* has been demonstrated to be associated with significantly improved survival in patients with GBM treated with TMZ^4,5,19,20^. However, this conclusion has been based mainly on cohorts dominated by *IDH*-wildtype patients with GBM. Study of the roles of *MGMT* promoter methylation in a homogeneous cohort of *IDH*-mutant patients with GBM is therefore urgently needed.

The methylation status of the *MGMT* promoter is widely classified as “methylated” or “unmethylated” through quantitative methods such as pyrosequencing (PSQ) with a defined cutoff value^21–24^. How the optimal cutoff value should be defined and whether a single cutoff value can fully reflect the clinical response to TMZ therapy are critical issues remaining to be resolved^24–26^. Several studies in cohorts dominated by *IDH*-wildtype patients with GBM or comprising exclusively *IDH*-wildtype patients with GBM have shown that the survival of TMZ-treated patients with GBM can be divided into 3 or more groups on the basis of the extent of *MGMT* promoter methylation^27–29^. In addition, the cutoff value determined in *IDH*-wildtype GBM cases might not be suitable for *IDH*-mutant cases. Considering gliomas overall, the *MGMT* promoter methylation of *IDH*-mutant glioma (mainly lower-grade glioma, WHO grade II/III) is significantly higher than that of *IDH*-wildtype glioma (mainly GBM), and more than 90% of cases of *IDH*-mutant gliomas have been determined to be *MGMT* promoter methylated according to the cutoff value used for *IDH*-wildtype GBM^2,23,30^. Methylation levels of the *MGMT* promoter can be used to stratify the progression-free survival (PFS) of TMZ-treated *IDH*-mutant lower-grade glioma (LGG) with TMZ therapy into 3 groups according to cutoff values significantly higher than those commonly used in *IDH*-wildtype GBM cases^30^. Together, these findings suggest that the predictive cutoff value for *MGMT* promoter methylation in *IDH*-mutant GBM must be reassessed because it is likely to differ from that in *IDH*-wildtype GBM.

Here, our aim was to determine the predictive value of *MGMT* promoter methylation levels in *IDH*-mutant GBM. We investigated the effects of *IDH* mutant status on *MGMT* methylation and MGMT mRNA expression in 187 IDH-mutant GBM and 173* IDH*-wildtype cases. Then, we compared the PFS and overall survival (OS) of patients in different methylation groups of 75 TMZ treated *IDH*-mutant GBM cases. We additionally compared the predictive cutoff levels of MGMT promoter PSQ testing between* IDH*-mutant and *IDH*-wildtype GBM samples.

## Materials and methods

### Samples, clinical and patient data

A total of 187 patients diagnosed between 2006 and 2018 (WHO grade IV) with GBM with *IDH* mutation were enrolled in the Chinese Glioma Genome Atlas (CGGA) Database. Another 173 patients diagnosed with GBM with *IDH-*wildtype for whom *MGMT* promoter methylation information was available were also enrolled for comparison. The clinical characteristics of these patients are summarized in **Supplementary Table S1**.

There are 98 CpG sites located in the *MGMT* promoter region (chr10: 131264949–131265710 from the 5′-end to the 3′-end). Our previous study showed that the average methylation levels at 4 or more of CpG sites 72–82 have similar predictive effects^2,31^. In 50 patients who were diagnosed before June 2016 with *IDH*-mutant GBM and treated with TMZ for at least 3 cycles, methylation information for CpG sites 75–78 was available (cohort A); in 25 additional cases diagnosed after June 2016, methylation information for CpG sites 76–79 was available (cohort B). These data were used to study the predictive value of *MGMT* promoter methylation levels for TMZ treatment. The TMZ protocols for these patients followed the Chinese Glioma Cooperative Group Clinical Practice Guidelines for the management of adult diffuse gliomas^1^. For patients with primary GBM or recurrent/secondary GBM who had not received radiotherapy, the chemotherapy regimen was TMZ at a daily dose of 75 mg/m^2^ during concurrent chemoradiotherapy, and then at least 3 adjuvant TMZ treatment cycles over 5 days during each 28-day cycle at doses of 150–200 mg/m^2^. Patients with recurrent/secondary GBM who had previously received radiotherapy received only TMZ treatment at a dose of 150–200 mg TMZ mg/m^2^ over 5 days during each 28-day cycle for at least 3 cycles.

The PFS and OS information for all cases was extracted from the CGGA database. We also compared the clinical characteristics of cases in cohort A and cohort B (**Table 1**). PFS was determined on the basis of RANO criteria^32^, and the OS and PFS of patients with recurrent/secondary were calculated from the date of the recurrent/secondary diagnosis.

**Table 1:** Characteristics of *IDH*-mutant patients with GBM used in survival analysis

	Cohort A (CpGs 75–78, *n* = 50)		Cohort B (CpGs 76–79, *n* = 25)		*P*
Median age (range)	41 (26–63)	43 (29–66)	0.3887^a^		
Gender					1.0000^b^
Male	30	60.0%	15	60.0%	
Female	20	40.0%	10	40.0%	
Type					0.4091^b^
Primary	23	46.0%	9	36.0%	
Recurrent/secondary	27	54.0%	16	64.0%	
Resection					0.0228^b^
Gross total	33	66.0%	8	32.0%	
Subtotal	16	32.0%	13	52.0%	
Unknown	1	2.0%	4	16.0%	
Median KPS (range)	70 (50–90)		70 (50–90)		0.5464^b^
<70	17	34.0%	9	36.0%	
≥70	22	44.0%	16	64.0%	
Unknown	11	22.0%	0	0.0%	
TMZ cycles					0.8511^b^
≥3 and <6	13	26.0%	6	24.0%	
≥6	37	74.0%	19	76.0%	
MGMT promoter methylation					0.5431^b^
≥30%	14	28.0%	11	44.0%	
≥20%, <30%	8	16.0%	4	16.0%	
≥10%, <20%	16	32.0%	6	24.0%	
<10%	12	24.0%	4	16.0%	
Median PFS (months)	10.57		8.32		0.5711^c^
Median OS	16.13		13.2		0.3240^c^

^a^Calculated by the nonparametric test; ^b^Calculated by the chi-square test; ^c^Calculated by the log-rank test in Kaplan-Meier curves.

The tumor histological grades for all patients in this study were determined independently by 2 pathologists. All specimens with > 80% tumor cells were used to determine *MGMT* promoter methylation by PSQ. The *IDH1* R132H and *IDH2* R172K/M mutations were determined by whole-exome sequencing or PSQ, as previously reported^2,31,33^. *MGMT* mRNA expression data were obtained by RNA sequencing with the Illumina HiSeq 2000 platform (Illumina, San Diego, CA, USA) as previously reported^31,34,35^.

### Ethical approval

This study was approved by the Beijing Tiantan Hospital institutional review board (Approval No. KY2014-002-02). All patients in this study were enrolled in the CGGA program (KY2014-002-02), and informed consent was obtained from each patient involved in our research.

### Pyrosequencing of MGMT promoter methylation

The PSQ testing of *MGMT* promoter methylation was performed as previously reported^2^. Briefly, DNA was extracted in formalin-fixed paraffin-embedded samples with a QIAamp DNA FFPE Tissue Kit (Qiagen, Hilden, Germany). Then 100 ng DNA was bisulfite converted with an Epitect Bisulfite kit (Qiagen, Hilden, Germany) according to the manufacturer’s protocol. The bisulfite-treated DNA was amplified and then sequenced by PSQ. The amplification primers were the forward primer 5′-GTT TYG GAT ATG TTG GGA TAG TT-3′ and the biotinylated reverse primer 5′-biotin-ACR ACC CAA ACA CTC ACC AA-3′. The methylation levels of CpG sites 75–78 were obtained with the PSQ sequencing primers 5′-GAT ATG TTG GGA TAG T-3′ or 5′-GTT TTT AGA AYG TTT TG-3′. The methylation levels of CpG sites 76–79 were detected with a commercial *MGMT* PSQ kit (Qiagen, Hilden, Germany) with a PyroMark Q24 System (Qiagen, Hilden, Germany). Standardized positive and negative controls were included in all routine PSQ testing, and every PSQ test was performed by 2 experienced molecular neuropathologists together.

### Statistical analysis

Statistical analysis was performed in GraphPad Prism 7 (GraphPad Software, California, USA) and SPSS (IBM, NY, USA). A *P*-value of less than 0.05 was considered significant in this study. A nonparametric test was used to compare the age distribution between the 2 subgroups; two-tailed Student’s t-test was used to compare the *MGMT* mRNA expression between the 2 subgroups; and χ^2^ tests were used to compare the distribution of *MGMT* promoter methylation statuses and other clinicopathological features. The Kaplan-Meier method with log-rank test was used to compare the PFS and OS of patients in different subgroups. Univariate and multivariate survival analyses were performed with the Cox regression model to study the survival associations of different candidate factors.

## Results

### Cases and clinical features

**Figure 1:**
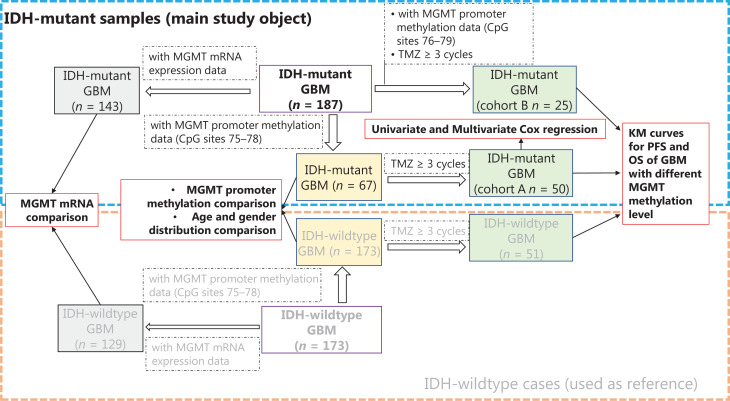
The workflow and sample selection criteria of this study.

The clinical implications of *MGMT* promoter methylation have been extensively studied in *IDH*-wildtype or *IDH*-heterogeneous cohorts. Therefore, we mainly focused on *IDH*-mutant cases and used *IDH*-wildtype cases as a comparison reference (**Figure 1**). A group of *IDH*-mutant GBM cases (*n* = 75) with methylation information from *MGMT* promoter CpG sites 75–78 was selected and compared with *IDH*-wildtype GBM cases (*n* = 173) for *MGMT* promoter methylation, age, and gender distribution. Among these 75 *IDH*-mutant cases, a group of cases (*n* = 50) receiving at least 3 cycles of TMZ treatment was used as cohort A to study the predictive value of *MGMT* promoter methylation levels in *IDH*-mutant GBM, and a group of *IDH*-wildtype cases (*n* = 99) was used as a comparison reference. Next, another cohort (cohort B) of 25 *IDH*-mutant cases receiving at least 3 cycles of TMZ, for which methylation information for *MGMT* promoter CpG sites 76–79 was available, was selected to validate the predictive value of *MGMT* promoter methylation. The clinical characteristics of cohorts A and B are summarized and compared in **Table 1**. Moreover, we compared the *MGMT* mRNA expression levels between *IDH*-mutant (*n* = 143) and *IDH*-wildtype (*n* = 129) cases. The clinical characteristics of all cases in this study are summarized in **Supplementary Table S1**.

### The effects of IDH mutation on MGMT promoter methylation in GBM

**Figure 2:**
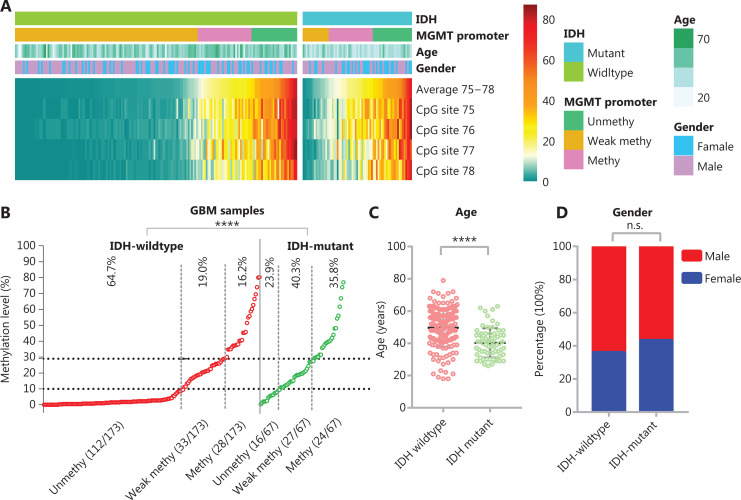
The effect of *IDH* mutation on *MGMT* promoter methylation in GBM. (A) Heatmap showing the methylation levels of CpG sites 75–78 in the *MGMT* promoter in GBM samples with different *IDH* mutant status. For the *MGMT* promoter, the average methylation level of CpG sites 75–78 is denoted unmethy (unmethylated), <10%; weak methy (methylated), ≥10% and <30%; or methy, ≥30%. (B) The distribution of average methylation levels of CpG sites 75–78 was compared between *IDH*-wildtype and *IDH*-mutant GBM. ****P < 0.0001 calculated by the chi-square test. (C) The age of *IDH*-wildtype and *IDH*-mutant patients with GBM was compared. ****P < 0.0001 calculated by the nonparametric test. (D) The gender distribution was compared between *IDH*-wildtype and *IDH*-mutant patients with GBM.

The methylation levels of CpG sites 75–78 are presented as a heatmap for *IDH*-wildtype and *IDH*-mutant GBM cases (**Figure 2A**). For *IDH*-wildtype GBM, a cutoff of ≥10% is usually used to define a “methylated” or “unmethylated” *MGMT* promoter according to the average methylation level, and a cutoff ≥30% is recommended to stratify a “weakly methylated” *vs* “methylated” promoters^28,31^. In our cohorts, we also observed that the OS and FPS of cases in the “weakly methylated” (≥10%, <30%) group differed from that of cases in the “unmethylated” (>10%) and “methylated” (≥30%) groups (**Supplementary Figure S1**). Thus, we divided the *MGMT* promoter methylation status into 3 levels in the heatmap: “unmethylated,” “weakly methylated,” and “methylated.” The proportion of unmethylated *MGMT* promoter cases in *IDH*-mutant GBM was significantly lower (23.9% *vs.* 64.7%) than that in *IDH*-wildtype GBM, whereas the proportion of methylated *MGMT* promoter cases in *IDH*-mutant GBM (35.8%) was similar to the sum of the proportions of weakly methylated and methylated *MGMT* promoter cases for *IDH*-wildtype GBM (35.2%) (**Figure 2B**). In agreement with the literature^10,13^, the patients with *IDH*-mutant GBM were significantly younger at diagnosis than *IDH*-wildtype patients with GBM at diagnosis (**Figure 2C**). There was no difference in the gender distributions of patients with *IDH*-mutant GBM and *IDH*-wildtype GBM (**Figure 2D**).

*MGMT* promoter methylation is negatively correlated with *MGMT* mRNA expression in *IDH*-wildtype or *IDH*-heterogeneous cases^30,31,36^. We also found that the expression of *MGMT* mRNA in *IDH*-mutant GBM (*n* = 143) was significantly lower (*P* < 0.0001) than that of *IDH*-wildtype GBM (*n* = 129) (**Supplementary Figure S2A** and **S2B**). In addition, we observed a negative correlation between *MGMT* mRNA expression and *MGMT* promoter methylation (averaged over CpG sites 75–78) in 41 *IDH*-mutant GBM cases, and significantly lower expression of *MGMT* mRNA in cases with higher (≥30%) *MGMT* promoter methylation (**Supplementary Figure S3**).

### The predictive value of MGMT promoter methylation in TMZ-treated IDH-mutant GBM

**Figure 3:**
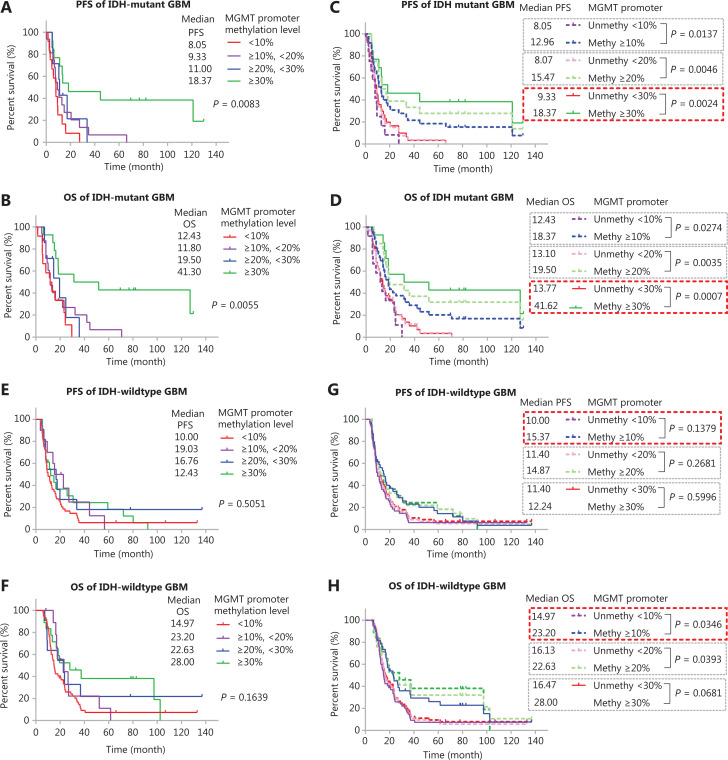
Survival analysis of *IDH*-mutant GBM with different *MGMT* promoter methylation levels. (A, B) Kaplan-Meier curves for PFS and OS of *IDH*-mutant patients with GBM in different methylation groups. (C, D) Kaplan-Meier curves for PFS and OS of *IDH*-mutant patients with GBM stratified by different cutoff values. (E, F) Kaplan-Meier curves for PFS and OS of *IDH*-wildtype patients with GBM in different methylation groups. (G, H) Kaplan-Meier curves for PFS and OS of *IDH*-wildtype patients with GBM stratified by different cutoff values. *P*-value calculated by the log-rank test. *MGMT* promoter methylation levels were calculated on the basis of the average methylation levels of CpG sites 75–78.

We used Kaplan-Meier curves to compare the PFS and OS of TMZ-treated* IDH*-mutant patients with GBM with different methylation levels: <10%, ≥10% and <20%, ≥20% and <30%, and ≥30% (**Figure 3A** and **3B**). The median PFS in months was 8.05 (<10%), 9.33 (≥10% and <20%), 11.00 (≥20% and <30%), and 18.37 (≥30%) (**Figure 3A**). The median OS in months was 12.43 (<10%), 11.80 (≥10% and <20%), 19.50 (≥20% and <30%), and 41.30 (≥30%) (**Figure 3B**). We also compared the PFS and OS of patients stratified by different cutoff values: ≥10%, ≥20%, and ≥30% (**Figure 3C** and **3D**). Although these cutoff values were able to stratify the OS and PFS of patients, the methylation status determined by a single high cutoff value (≥30%) had the best ability to stratify both PFS and OS. With this cutoff, the PFS of methylated cases (≥30%) was significantly longer, at 18.37 months (*P* = 0.0024) than that of the unmethylated group (<30%), at 9.33 months; in addition, the OS of methylated cases, at 41.62 months, was significantly longer (*P* = 0.0007) than that of the unmethylated cases, at 13.77 months.

We also analyzed the PFS and OS in different groups of TMZ-treated *IDH*-wildtype GBM samples (**Figure 3E** and **3F**). The median PFS in months was 10.00, 19.03, 16.76, and 12.43 months for methylation levels <10%, 10%–20%, 20%–30%, and >30%, respectively. The median OS in months was 14.97, 23.20, 22.63, and 28.00 for methylation levels <10%, 10%–20%, 20%–30%, and >30%, respectively. These results differed from those in *IDH*-mutant GBM cases, and the survival of patients was similar among different groups with methylation ≥10%. None of the cutoff values (≥10%, ≥20%, and ≥30%) stratified the PFS of patients (**Figure 3G**), and the cutoff ≥10% showed the lowest *P* value (*P* = 0.1397). A cutoff of ≥10% and ≥20% but not ≥30% significantly stratified the OS of patients, and a cutoff ≥10% showed a lower *P* value (*P* = 0.0346 *vs.* 0.0393) (**Figure 3H**).

The above findings suggested that *MGMT* promoter methylation has predictive value for both *IDH*-mutant and *IDH*-wildtype GBM cases, but the optimal cutoff value for *IDH*-mutant GBM is higher than that for *IDH*-wildtype GBM. To determine whether the *MGMT* promoter status (determined by cutoff ≥30%) might correlated with other clinical features in *IDH*-mutant GBM, we compared the clinical features between cases with or without *MGMT* promoter methylation in cohort A. No significant difference was observed between unmethylated and methylated cases in terms of age, gender distribution, tumor type (primary or recurrent/secondary), extent of resection, KPS scores, or TMZ cycles (**Table 2**). Univariate and multivariate Cox regression analyses were performed to determine whether the methylation status with a cutoff ≥30% was independently associated with the OS of *IDH*-mutant GBM cases in cohort A. Univariate Cox analysis indicated that *MGMT* promoter methylation status [unmethylated *vs.* methylated, *P* = 0.001, HR (hazard ratio) = 3.691 (1.689–8.063)] and tumor type [primary *vs.* recurrent/secondary, *P* = 0.005, HR = 0.366 (0.182–0.733)], but not age, gender, or extent of resection, significantly correlated with OS (**Table 3**). In the multivariate Cox analysis, *MGMT* promoter methylation status [unmethylated *vs.* methylated, *P* = 0.002, HR = 3.560 (1.600–7.920)] and tumor type [primary *vs.* recurrent/secondary, *P* = 0.010, HR = 0.384 (0.186–0.794)] remained significantly correlated with OS (**Table 3**).

**Table 2:** Comparison of characteristics of *IDH*-mutant GBM samples with or without *MGMT* methylation (cutoff ≥30%) in cohort A

	Unmethylated (*n* = 35)		Methylated (*n* = 15)		*P*
Median age (range)	41 (26–63)		39 (33–62)		0.4529^a^
Gender					0.2077^b^
Male	23	65.7%	7	46.7%	
Female	12	34.3%	8	53.3%	
Type					0.1935^b^
Primary	14	40.0%	9	60.0%	
Recurrent	21	60.0%	6	40.0%	
Resection					0.1053^b^
Gross total	26	74.3%	8	53.3%	
Subtotal	8	22.9%	7	46.7%	
Unknown	1	2.9%	0	0.0%	
TMZ cycles					0.9706^b^
≥3 and <6	9	25.7%	4	26.7%	
≥6	30	85.7%	13	86.7%	
KPS					0.6479^b^
<70	12	34.3%	5	33.3%	
≥70	14	40.0%	8	53.3%	
Unknown	9	25.7%	2	13.3%	
Median PFS (months)	9.33		26.04		0.0012^c^
Median OS (months)	13.1	35.8	0.0004^c^		

^a^Calculated by the nonparametric test; ^b^Calculated by the chi-square test; ^c^Calculated by the log-rank test in Kaplan-Meier curves.

**Table 3:** Univariate and multivariate Cox regression analysis in cohort A

	Univariate Cox analysis				Multivariate Cox analysis			
	*P*	HR	95% CI for HR		*P*	HR	95% CI for HR	
			Lower	Higher			Lower	Higher
Age	0.761	0.995	0.963	1.028	–	–	–	–
Gender (female *vs.* male)	0.223	0.663	0.343	1.283	–	–	–	–
MGMT (unmethy *vs.* methy)	0.001	3.691	1.689	8.063	0.002	3.560	1.600	7.920
Extent of resection (total *vs.* subtotal)	0.470	1.287	0.649	2.549	–	–	–	–
Type (primary *vs.* recurrent/secondary)	0.005	0.366	0.182	0.733	0.010	0.384	0.186	0.794

HR, hazard ratio; CI, confidence interval; methy, methylated; unmethy, unmethylated.

Therefore, we further analyzed the predictive value of* MGMT* promoter methylation status (cutoff ≥30%) in primary and recurrent/secondary GBM cases. The results revealed that *MGMT* promoter methylation significantly stratified PFS and OS in both the primary (**Supplementary Figure S4A** and **S4B**) and recurrent/secondary (**Supplementary Figure S4C** and **S4D**) GBM cases. In the primary *IDH*-mutant GBM cases, the median PFS in months was 14.90 (unmethylated, <30%) and 82.90 (methylated, ≥30%), and the median OS in months was 19.50 (unmethylated, <30%) and 127.10 (methylated, ≥30%). In the recurrent/secondary cases, the median PFS in months was 7.17 (unmethylated, <30%) and 13.40 (methylated, ≥30%), and the median OS in months was 10.64 (unmethylated, <30%) and 18.43 (methylated, ≥30%). These findings suggest that the predictive value of *MGMT* methylation status is independent of age, gender, extent of resection, and tumor type (primary or recurrent/secondary) in *IDH*-mutant GBM.

### Validation of the predictive value of MGMT promoter methylation in another cohort of IDH-mutant GBM cases

**Figure 4:**
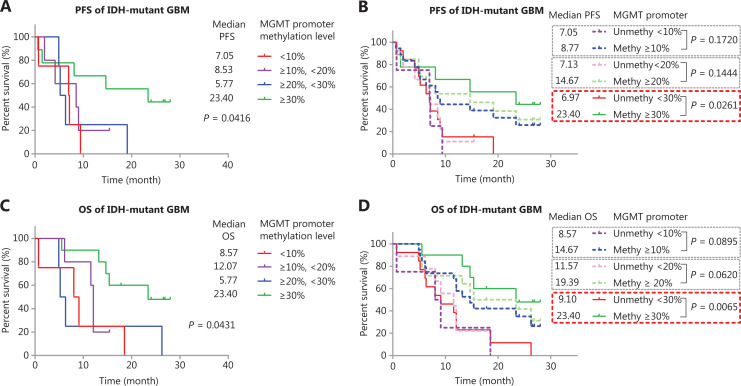
Validation of the predictive value of *MGMT* promoter methylation in another cohort of TMZ treated *IDH*-mutant GBM. (A) Kaplan-Meier curves for PFS of *IDH*-mutant patients with GBM in different methylation groups. (B) Kaplan-Meier curves for PFS of *IDH*-mutant patients with GBM, stratified by different cutoff values. (C) Kaplan-Meier curves for OS of *IDH*-mutant patients with GBM in different methylation groups. (D) Kaplan-Meier curves for OS of *IDH*-mutant patients with GBM, stratified by different cutoff values. *P*-value calculated by the log-rank test. *MGMT* promoter methylation levels were calculated on the basis of the average methylation levels of CpG sites 76–79.

We sought to validate the predictive value of *MGMT* promoter methylation in *IDH*-mutant GBM as well as the use of a higher *MGMT* promoter methylation cutoff value (such as ≥30%) for *IDH*-mutant GBM. We further compared PFS and OS in different groups in another 25 *IDH*-mutant GBM cases diagnosed after June 2016, with the *MGMT* promoter methylation levels calculated by using CpG sites 76–79 (cohort B) (**Figure 4**). The median PFS of cases with methylation ≥30% was 23.40 months, which was significantly longer than that in other groups: 7.05 (<10%), 8.53 (≥10% and <20%), and 5.77 (≥20% and <30%) (**Figure 4A**). Only the methylation status determined by a high cutoff value (≥30%) significantly stratified (*P* = 0.0261) the PFS of patients (**Figure 4B**), possibly because of the limited number of cases in cohort B. A similar result was observed in the OS of patients: the median OS in months was 8.57 (<10%), 12.03 (≥10% and <20%), 5.77 (≥20% and <30%), and 23.40 (≥30%). Only the methylation status determined by a high cutoff value (≥30%) significantly stratified (*P* = 0.0065) the OS of patients (**Figure 4C** and **4D**).

## Discussion

*MGMT* promoter methylation is widely used a biomarker to predict which patients with GBM will benefit from TMZ treatment^5,25,29,37–39^. According to the most recent WHO classification, GBM should be further classified as *IDH*-wildtype or *IDH*-mutant GBM, which have different genetic, epigenetic, and transcriptional characteristics^6,9,14,40^. In this study, we further confirmed that, compared with *IDH*-wildtype GBM cases, *IDH*-mutant GBM cases showed significantly higher MGMT promoter methylation but significantly lower *MGMT* mRNA expression. This finding is consistent with previous reports indicating that most *IDH*-mutant GBM cases are *MGMT* promoter methylated, according to the commonly used cutoff value for *IDH*-wildtype GBM^23,30^. Therefore, we showed that the extent of *MGMT* promoter methylation also had predictive value in *IDH*-mutant GBM receiving TMZ therapy, and that the optimal cutoff value of *MGMT* promoter methylation should be dramatically higher than that used for *IDH*-wildtype cases.

The effect of *IDH* mutation on methylation of the *MGMT* promoter has been shown in LGG, and the methylation level of the *MGMT* promoter is significantly elevated in *IDH*-mutant LGG^30^. Here, we also demonstrated an elevation in *MGMT* methylation levels in *IDH*-mutant GBM cases. A previous study has reported that *IDH-*mutant status and *MGMT* promoter methylation status appear to be gender related, and that the methylated* MGMT* promoter is found more frequently in females, whereas *IDH* mutation is more often detected in males^41^. In the cohorts in this study, there was no significant difference in the gender distribution between *IDH*-mutant and *IDH*-wildtype GBM cases, thus suggesting that the differential methylation levels of *MGMT* promoters were indeed caused by different *IDH*-mutant statuses.

The methylation status of the *MGMT* promoter is widely classified as “methylated” or “unmethylated” through gel-based methylation-specific polymerase chain reaction or quantitative methods with determined cutoff values^26^. Scientific community consensus is lacking regarding how to determine the cutoff values for the quantitative detection methods of *MGMT* promoter methylation, including PSQ, semi-quantitative methylation-specific polymerase chain reaction, and methylation chip assay^3,24,26,28^. There are 98 CpG sites in the *MGMT* promoter region, and the methylation levels of CpG sites 72–90, localized in exon 1 and the subsequent intron 1, are thought to be most negatively correlated with *MGMT* expression^42^. We have reported that combinations of methylation at 4 or more CpG sites have equivalent predictive value for *MGMT* expression in gliomas and TMZ therapy response in GBM^31^. The high heterogeneity of each CpG site and the different principles of each test method yielding cutoff values should be determined and validated in different assays^3,29^. However, the use of a single invariant cutoff value is increasingly challenged in the quantitative detection of *MGMT* methylation, and determining “unmethylated” status and a potential “grey zone” through 2 cutoff values has been suggested^3,27–29^. Here, to study the predictive value of *MGMT* promoter methylation in *IDH*-mutant GBM more objectively, we also divided the cases into 3 methylation groups according to 2 reported cutoff values in GBM. We observed an overlap between the survival curves of cases with weakly methylated (10%–30%) and unmethylated (<10%) *MGMT* promoter in *IDH*-wildtype GBM. For *IDH*-mutant GBM, we found that ≥30% is a feasible cutoff for PSQ testing of *MGMT* promoter methylation, and we validated the predictive value in another cohort with different CpG sites tested. However, the determination of the optimal cutoff value and whether a “gray zone” interval exists remains to be determined in the future through study of a larger number of samples.

The predictive value of *MGMT* promoter methylation for the response to TMZ treatment could also be affected by other molecular characteristics, including chromosome variation, DNA alterations, RNA expression profiles, and even the immune microenvironment^15,34,43–48^. Recently, the critical role of noncoding RNA in regulating MGMT expression and TMZ-sensitivity has also been revealed^43,44^. Given the different molecular and RNA expression features existing among various subtypes of gliomas, the predictive value of *MGMT* promoter methylation in other types of glioma remains controversial, possibly because the cutoff value used in *IDH*-wildtype GBM has also been used to assess the role of *MGMT* promoter methylation in other types of glioma^25,26,30,38,49^. In either PSQ or methylation chip assays, the *MGMT* promoters of most *IDH* mutant adult LGGs are interpreted as methylated on the basis of the cutoff value used for GBM (with most *IDH*-wildtype cases)^23,38,49^. We believe that specific cutoff values should be determined for each homogeneous glioma group, because the molecular characteristics of glioma differ in different pathological groups^1,6,9,14^. In a retrospective study of EORTC-22033 randomized phase III trial samples, *MGMT* promoter methylation was revealed to have predictive value in *IDH*-mutant grade II glioma treated with TMZ, but the cutoff value used in that study was dramatically higher than that commonly used in *IDH*-wildtype GBM^30^. Here, we also found that the cutoff of ≥10%, which is commonly used in *IDH*-wildtype GBM PSQ testing, was not suitable for *IDH*-mutant GBM, probably as a consequence of the differing molecular features, such as chromosome 10 loss, between *IDH*-mutant and *IDH*-wildtype GBM^2,29^.

Initially, the predictive value of *MGMT* promoter methylation was focused on primary GBM cases. Recently, a study with a large cohort of matched primary and recurrent *IDH*-wildtype GBM has shown that *MGMT* promoter methylation status differs between primary and recurrent tumors, and *MGMT* methylation status remains predictive for TMZ response in tumor recurrence^37^. Because of the low frequency of *IDH*-mutant GBM, whether the methylation characteristic of recurrent/secondary *IDH*-mutant GBM could be classified into the same group as primary *IDH*-mutant GBM is unclear^50^. We included both primary and recurrent/secondary *IDH*-mutant GBM in our cohort, and we demonstrated the predictive value of *MGMT* promoter methylation in both. In addition, multivariate Cox regression confirmed the independent association between *MGMT* promoter methylation status and OS of *IDH*-mutant patients with GBM treated by TMZ. These findings support the need to re-test *MGMT* promoter methylation status in recurrent/secondary *IDH*-mutant GBM.

Because this was a retrospective study with cases from a single center, some limitations restrict the interpretation of our data. Nevertheless, we characterized the predictive value of *MGMT* promoter methylation in a relatively large number of *IDH*-mutant GBM cases whose clinical features are well documented.

## Conclusions

In conclusion, we demonstrate that the extent of *MGMT* promoter methylation has predictive value in both primary and recurrent/secondary *IDH* mutant GBM. We also recommend the use of higher cutoff value, such as ≥30% in PSQ testing, to interpret *MGMT* promoter methylation results in *IDH*-mutant GBM.

## References

[1] Jiang T, Mao Y, Ma W, Mao Q, You Y, Yang X (2016). CGCG clinical practice guidelines for the management of adult diffuse gliomas. Cancer Lett.

[2] Chai RC, Liu YQ, Zhang KN, Wu F, Zhao Z, Wang KY (2019). A novel analytical model of MGMT methylation pyrosequencing offers improved predictive performance in patients with gliomas. Mod Pathol.

[3] Chai RC, Chang YZ, Wang QW, Zhang KN, Li JJ, Huang H (2019). A novel DNA methylation-based signature can predict the responses of MGMT promoter unmethylated glioblastomas to temozolomide. Front Genet.

[4] Esteller M, Garcia-Foncillas J, Andion E, Goodman SN, Hidalgo OF, Vanaclocha V (2000). Inactivation of the DNA-repair gene MGMT and the clinical response of gliomas to alkylating agents. N Engl J Med.

[5] Hegi ME, Diserens AC, Gorlia T, Hamou MF, de Tribolet N, Weller M (2005). MGMT gene silencing and benefit from temozolomide in glioblastoma. N Engl J Med.

[6] Louis DN, Perry A, Reifenberger G, von Deimling A, Figarella-Branger D, Cavenee WK (2016). The 2016 World Health Organization Classification of Tumors of the Central Nervous System: a summary. Acta Neuropathol.

[7] Cao M, Cai J, Yuan Y, Shi Y, Wu H, Liu Q (2019). A four-gene signature-derived risk score for glioblastoma: prospects for prognostic and response predictive analyses. Cancer Biol Med.

[8] Li WB, Tang K, Chen Q, Li S, Qiu XG, Li SW (2012). MRI manifestions correlate with survival of glioblastoma multiforme patients. Cancer Biol Med.

[9] Chai R, Zhang K, Wang K, Li G, Huang R, Zhao Z (2018). A novel gene signature based on five glioblastoma stem-like cell relevant genes predicts the survival of primary glioblastoma. J Cancer Res Clin Oncol.

[10] Yan H, Parsons DW, Jin G, McLendon R, Rasheed BA, Yuan W (2009). IDH1 and IDH2 mutations in gliomas. N Engl J Med.

[11] Brat DJ, Aldape K, Colman H, Figrarella-Branger D, Fuller GN, Giannini C (2020). cIMPACT-NOW update 5: recommended grading criteria and terminologies for IDH-mutant astrocytomas. Acta Neuropathol.

[12] Brat DJ, Aldape K, Colman H, Holland EC, Louis DN, Jenkins RB (2018). cIMPACT-NOW update 3: recommended diagnostic criteria for “Diffuse astrocytic glioma, IDH-wildtype, with molecular features of glioblastoma, WHO grade IV”. Acta Neuropathol.

[13] Hu H, Mu Q, Bao Z, Chen Y, Liu Y, Chen J (2018). Mutational landscape of secondary glioblastoma guides MET-targeted trial in brain tumor. Cell.

[14] Turcan S, Rohle D, Goenka A, Walsh LA, Fang F, Yilmaz E (2012). IDH1 mutation is sufficient to establish the glioma hypermethylator phenotype. Nature.

[15] Han S, Liu Y, Cai SJ, Qian M, Ding J, Larion M (2020). IDH mutation in glioma: molecular mechanisms and potential therapeutic targets. Br J Cancer.

[16] Yu D, Liu Y, Zhou Y, Ruiz-Rodado V, Larion M, Xu G (2020). Triptolide suppresses IDH1-mutated malignancy via Nrf2-driven glutathione metabolism. Proc Natl Acad Sci U S A.

[17] Shirahata M, Ono T, Stichel D, Schrimpf D, Reuss DE, Sahm F (2018). Novel, improved grading system(s) for IDH-mutant astrocytic gliomas. Acta Neuropathol.

[18] Gerson SL (2004). MGMT: its role in cancer aetiology and cancer therapeutics. Nat Rev Cancer.

[19] Hegi ME, Diserens AC, Godard S, Dietrich PY, Regli L, Ostermann S (2004). Clinical trial substantiates the predictive value of O-6-methylguanine-DNA methyltransferase promoter methylation in glioblastoma patients treated with temozolomide. Clin Cancer Res.

[20] Herrlinger U, Rieger J, Koch D, Loeser S, Blaschke B, Kortmann RD (2006). Phase II trial of lomustine plus temozolomide chemotherapy in addition to radiotherapy in newly diagnosed glioblastoma: UKT-03. J Clin Oncol.

[21] Quillien V, Lavenu A, Sanson M, Legrain M, Dubus P, Karayan-Tapon L (2014). Outcome-based determination of optimal pyrosequencing assay for MGMT methylation detection in glioblastoma patients. J Neurooncol.

[22] Brigliadori G, Foca F, Dall’Agata M, Rengucci C, Melegari E, Cerasoli S (2016). Defining the cutoff value of MGMT gene promoter methylation and its predictive capacity in glioblastoma. J Neurooncol.

[23] Mulholland S, Pearson DM, Hamoudi RA, Malley DS, Smith CM, Weaver JM (2012). MGMT CpG island is invariably methylated in adult astrocytic and oligodendroglial tumors with IDH1 or IDH2 mutations. Int J Cancer.

[24] Wick W, Weller M, van den Bent M, Sanson M, Weiler M, von Deimling A (2014). MGMT testing--the challenges for biomarker-based glioma treatment. Nat Rev Neurol.

[25] Weller M, Stupp R, Reifenberger G, Brandes AA, van den Bent MJ, Wick W (2010). MGMT promoter methylation in malignant gliomas: ready for personalized medicine. Nat Rev Neurol.

[26] Malmstrom A, Lysiak M, Kristensen BW, Hovey E, Henriksson R, Soderkvist P (2020). Do we really know who has an MGMT methylated glioma. Results of an international survey regarding use of MGMT analyses for glioma. Neurooncol Pract.

[27] Dunn J, Baborie A, Alam F, Joyce K, Moxham M, Sibson R (2009). Extent of MGMT promoter methylation correlates with outcome in glioblastomas given temozolomide and radiotherapy. Br J Cancer.

[28] Radke J, Koch A, Pritsch F, Schumann E, Misch M, Hempt C (2019). Predictive MGMT status in a homogeneous cohort of IDH wildtype glioblastoma patients. Acta Neuropathol Commun.

[29] Hegi ME, Genbrugge E, Gorlia T, Stupp R, Gilbert MR, Chinot OL (2019). MGMT promoter methylation cutoff with safety margin for selecting glioblastoma patients into trials omitting temozolomide: a pooled analysis of four clinical trials. Clin Cancer Res.

[30] Bady P, Kurscheid S, Delorenzi M, Gorlia T, van den Bent MJ, Hoang-Xuan K (2018). The DNA methylome of DDR genes and benefit from RT or TMZ in IDH mutant low-grade glioma treated in EORTC 22033. Acta Neuropathol.

[31] Chai RC, Zhang KN, Liu YQ, Wu F, Zhao Z, Wang KY (2019). Combinations of four or more CpGs methylation present equivalent predictive value for MGMT expression and temozolomide therapeutic prognosis in gliomas. CNS Neurosci Ther.

[32] Wen PY, Macdonald DR, Reardon DA, Cloughesy TF, Sorensen AG, Galanis E (2010). Updated Response Assessment Criteria for High-Grade Gliomas: Response Assessment in Neuro-Oncology Working Group. J Clin Oncol.

[33] Chai RC, Zhang YW, Liu YQ, Chang YZ, Pang B, Jiang T (2020). The molecular characteristics of spinal cord gliomas with or without H3 K27M mutation. Acta Neuropathol Commun.

[34] Chai RC, Wu F, Wang QX, Zhang S, Zhang KN, Liu YQ (2019). m(6)A RNA methylation regulators contribute to malignant progression and have clinical prognostic impact in gliomas. Aging (Albany NY).

[35] Liu YQ, Chai RC, Wang YZ, Wang Z, Liu X, Wu F (2019). Amino acid metabolism-related gene expression-based risk signature can better predict overall survival for glioma. Cancer Sci.

[36] Bady P, Sciuscio D, Diserens AC, Bloch J, van den Bent MJ, Marosi C (2012). MGMT methylation analysis of glioblastoma on the Infinium methylation BeadChip identifies two distinct CpG regions associated with gene silencing and outcome, yielding a prediction model for comparisons across datasets, tumor grades, and CIMP-status. Acta Neuropathol.

[37] Draaisma K, Chatzipli A, Taphoorn M, Kerkhof M, Weyerbrock A, Sanson M (2020). Molecular evolution of IDH wild-type glioblastomas treated with standard of care affects survival and design of precision medicine trials: a report From the EORTC 1542 study. J Clin Oncol.

[38] Wiestler B, Capper D, Hovestadt V, Sill M, Jones DT, Hartmann C (2014). Assessing CpG island methylator phenotype, 1p/19q codeletion, and MGMT promoter methylation from epigenome-wide data in the biomarker cohort of the NOA-04 trial. Neuro Oncol.

[39] Yamashita K, Hosoda K, Nishizawa N, Katoh H, Watanabe M (2018). Epigenetic biomarkers of promoter DNA methylation in the new era of cancer treatment. Cancer Sci.

[40] Hwang T, Mathios D, McDonald KL, Daris I, Park SH, Burger PC (2019). Integrative analysis of DNA methylation suggests down-regulation of oncogenic pathways and reduced somatic mutation rates in survival outliers of glioblastoma. Acta Neuropathol Commun.

[41] Matteoni S, Abbruzzese C, Villani V, Malorni W, Pace A, Matarrese P (2020). The influence of patient gender on clinical approaches to malignant glioma. Cancer Lett.

[42] Malley DS, Hamoudi RA, Kocialkowski S, Pearson DM, Collins VP, Ichimura K (2011). A distinct region of the MGMT CpG island critical for transcriptional regulation is preferentially methylated in glioblastoma cells and xenografts. Acta Neuropathol.

[43] Wu P, Cai J, Chen Q, Han B, Meng X, Li Y (2019). Lnc-TALC promotes O(6)-methylguanine-DNA methyltransferase expression via regulating the c-Met pathway by competitively binding with miR-20b-3p. Nat Commun.

[44] Li L, Wu P, Wang Z, Meng X, Zha C, Li Z (2020). NoncoRNA: a database of experimentally supported non-coding RNAs and drug targets in cancer. J Hematol Oncol.

[45] Zha C, Meng X, Li L, Mi S, Qian D, Li Z (2020). Neutrophil extracellular traps mediate the crosstalk between glioma progression and the tumor microenvironment via the HMGB1/RAGE/IL-8 axis. Cancer Biol Med.

[46] Wang Q, Cai J, Fang C, Yang C, Zhou J, Tan Y (2018). Mesenchymal glioblastoma constitutes a major ceRNA signature in the TGF-beta pathway. Theranostics.

[47] Li MY, Yang P, Liu YW, Zhang CB, Wang KY, Wang YY (2016). Low c-Met expression levels are prognostic for and predict the benefits of temozolomide chemotherapy in malignant gliomas. Sci Rep.

[48] Wang J, Cazzato E, Ladewig E, Frattini V, Rosenbloom DI, Zairis S (2016). Clonal evolution of glioblastoma under therapy. Nat Genet.

[49] Bell EH, Zhang P, Fisher BJ, Macdonald DR, McElroy JP, Lesser GJ (2018). Association of MGMT promoter methylation status with survival outcomes in patients with high-risk glioma treated with radiotherapy and temozolomide: an analysis from the NRG Oncology/RTOG 0424 trial. JAMA Oncol.

[50] Korshunov A, Casalini B, Chavez L, Hielscher T, Sill M, Ryzhova M (2019). Integrated molecular characterization of IDH-mutant glioblastomas. Neuropathol Appl Neurobiol.

