# Supplementary material for: Predictive value of MGMT promoter methylation on the survival of TMZ treated IDH-mutant glioblastoma
Source: Cancer Biol Med. 2021 Feb 15;18(1):271–82. doi: 10.20892/j.issn.2095-3941.2020.0179 (PMC7877176; doi:10.20892/j.issn.2095-3941.2020.0179)
Supplement: Supplementary file 1 [file cbm-18-271-s001.pdf]

Supplementary materials

Table S1 Characteristics of patients in this study

	IDH-mutant (*n* = 187)		*IDH*-wildtype (*n* = 173)	
Age (years)	41 (24–66)		52 (18–79)	
Gender				
Male	114	61.0%	111	64.2%
Female	73	39.0%	62	35.8%
Type				
Primary	59	31.6%	127	73.4%
Recurrent/secondary	128	68.4%	46	26.6%
Resection				
Gross total	119	63.6%	116	67.1%
Subtotal	60	32.1%	48	27.7%
Unknown	8	4.3%	9	5.2%
Radiotherapy				
Yes	84	44.9%	123	71.1%
No	85	45.5%	39	22.5%
Unknown	18	9.6%	11	6.4%
Chemotherapy (TMZ)				
Yes	130	69.5%	99	57.3%
No	37	19.8%	10	5.8%
Unknown	20	10.7%	64	36.9%
MGMT promoter methylation				
Methylated (≥30%)	36	19.3%	28	16.2%
Weakly methylated (≥10%, <30%)	40	21.4%	33	19.1%
Unmethylated (<10%)	22	11.8%	112	64.7%
Unknown	89	47.6%	0	0.0%
MGMT RNA expression				
Available	143	76.5%	129	74.6%
Unavailable	44	23.5%	44	25.4%

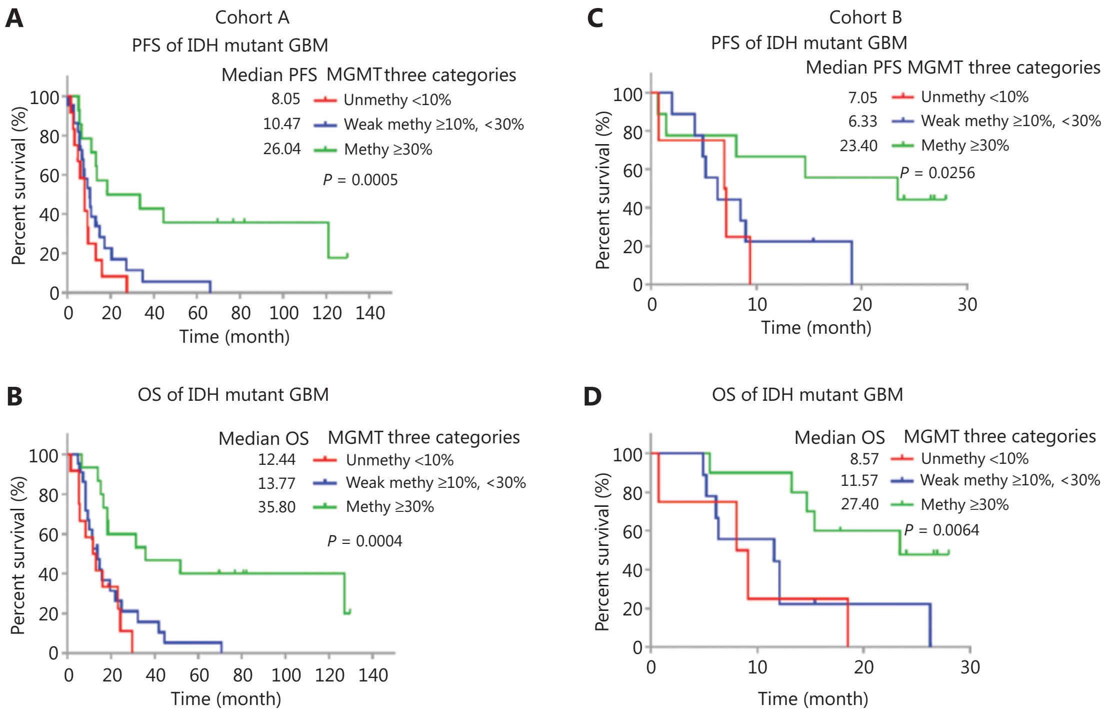

Figure S1 Survival analysis of TMZ-treated IDH-mutant GBM with different MGMT promoter methylation levels. (A, B) Kaplan-Meier curves for PFS and OS of *IDH*-mutant patients with GBM in different methylation groups in cohort A. *MGMT* promoter methylation levels were calculated by the average levels of CpG sites 75–78. (C, D) Kaplan-Meier curves for PFS and OS of IDH-mutant patients with GBM in different methylation groups in cohort In B, *MGMT* promoter methylation levels were calculated by the average levels of CpG sites 76–79. Unmethy: unmethylated, methy: methylated. *P*-value calculated by the log-rank test.

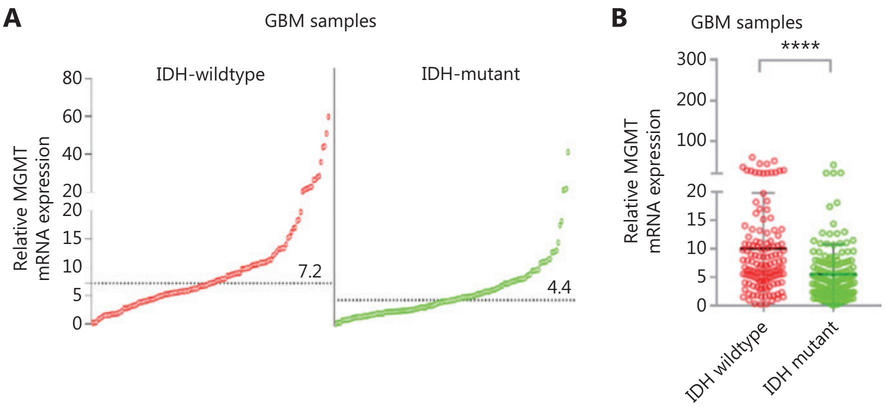

Figure S2 MGMT expression in GBM with or without *IDH* mutation. (A, B) MGMT mRNA expression levels were compared between GBM with (n = 143) or without (n = 129) *IDH* mutation. ****P < 0.0001 calculated by the t test.

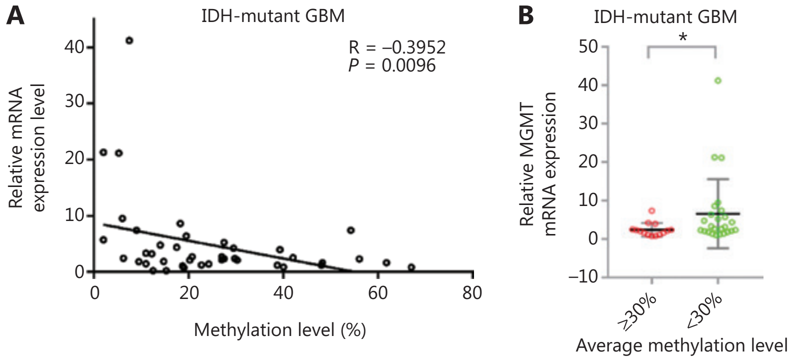

Figure S3 *MGMT* promoter methylation is negatively correlated with *MGMT* mRNA expression in *IDH*-mutant GBM. (A) Correlation of *MGMT* promoter methylation with *MGMT* mRNA expression in *IDH*-mutant GBM. R and *P*-value obtained by the Spearman R test. (B) MGMT mRNA expression levels were compared between *IDH*-mutant GBM with and without *MGMT* promoter methylation. *$P < 0.05$ calculated by t test.

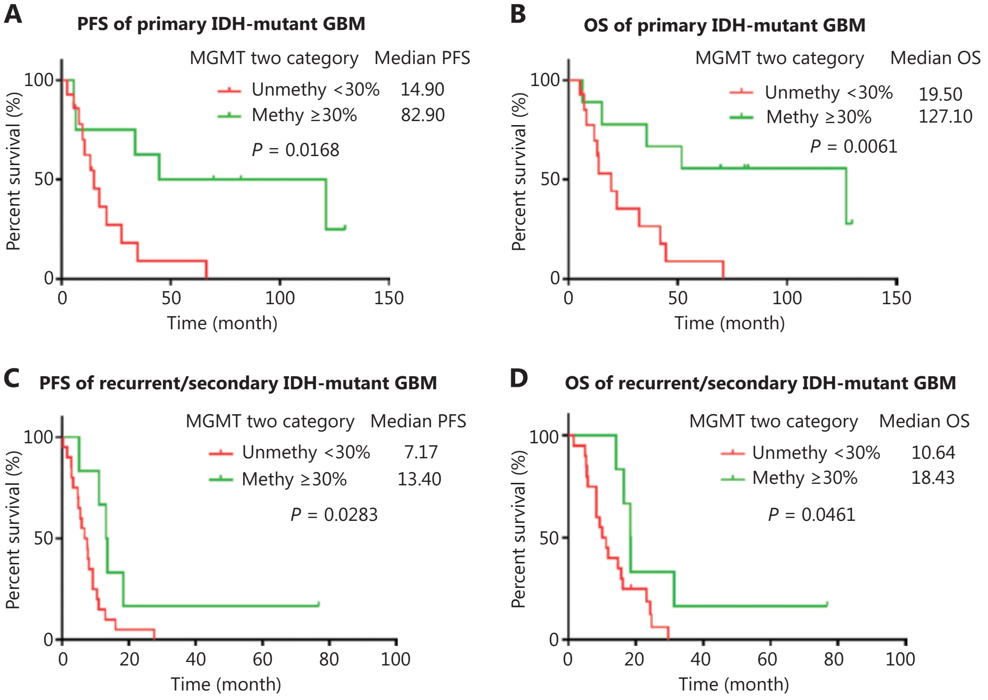

Figure S4 The predictive value of *MGMT* promoter methylation in primary and recurrent/secondary *IDH*-mutant GBM. (A, B) Kaplan-Meier curves for PFS and OS of primary *IDH*-mutant patients with GBM in different methylation groups. (C, D) Kaplan-Meier curves for PFS and OS of recurrent/secondary *IDH*-mutant patients with GBM in different methylation groups. Unmethy: unmethylated, methy: methylated. *P*-value calculated by log-rank test. *MGMT* promoter methylation levels were calculated by the average methylation levels of CpG sites 75–78.
